# Recent advances in transition-metal-free arylation reactions involving hypervalent iodine salts

**DOI:** 10.3762/bjoc.20.243

**Published:** 2024-11-13

**Authors:** Ritu Mamgain, Kokila Sakthivel, Fateh V Singh

**Affiliations:** 1 Department of Chemistry, SAS, Vellore Institute of Technology Chennai, Chennai-600 127, Tamil Nadu, India

**Keywords:** arylation reaction, diaryliodonium salts, electrophilic arylation reagent, metal-free arylation, rearrangement reaction

## Abstract

Diaryliodonium salts have become widely recognized as arylating agents in the last two decades. Both, symmetrical and unsymmetrical forms of these salts serve as effective electrophilic arylating reagents in various organic syntheses. The use of diaryliodoniums in C–C and carbon–heteroatom bond formations, particularly under metal-free conditions, has further enhanced the popularity of these reagents. In this review, we concentrate on various arylation reactions involving carbon and other heteroatoms, encompassing rearrangement reactions in the absence of any metal catalyst, and summarize advancements made in the last five years.

## Introduction

The chemistry of hypervalent iodine compounds is well-established and they are prevalent as oxidants and electrophilic reagents in organic conversions [1–3]. They have gained significant attention due to their high reactivity and ability to carry out various useful transformations under mild, eco-friendly reaction conditions [4–11]. Various review articles [12–26] and books [27–28] have appeared on the chemistry of hypervalent iodine compounds. In the past two decades, diaryliodonium salts (DAIS), a versatile category of hypervalent iodine compounds, have seen significant progress in hypervalent iodine chemistry. Their efficiency and environmentally friendly characteristics have positioned DAIS as next-generation arylation reagents [29–30]. Other than aromatic electrophiles in aryl-transfer processes, DIAS are frequently employed as photoinitiators for cationic polymerizations [31–33], Lewis acids [34], oxidants [35–36] and in the field of macromolecular chemistry [37–38]. Additionally, biological activity is also exhibited by iodonium salts, often due to their capability to function as radical initiators.

The use of diaryliodonium salts as efficient electrophilic arylating reagents in a wide range of organic transformations is due to their unique features such as solid-state nature, excellent stability, and the presence of a robust leaving group [39–42]. They offer several advantages over traditional reagents, including low toxicity, high reactivity, and excellent selectivity [43] under simple reaction conditions. The distinctive reactivity of DIAS enables the smooth arylation of various carbon and heteroatom nucleophiles under gentle conditions, with or without the use of transition metals [44]. Thus, they address both the financial and environmental challenges associated with organic synthesis by acting as environmentally benign substitutes for costly organometallic catalysts and heavy-metal-based oxidants.

**Figure 1:**
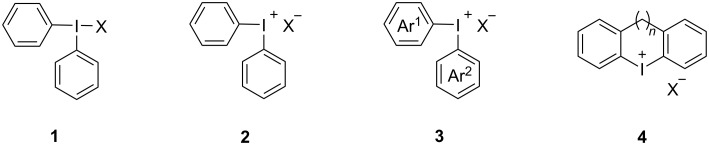
Various structures of iodonium salts.

Diaryl iodide salts consist of two aryl groups attached to an iodine atom and an associated "anion". X-ray studies revealed that these iodine(III) compounds typically have a T-type structure **1** (Figure 1). In solution, they dissociate into Ar_2_I^+^ and X^−^ counterions **2**, with the degree of dissociation influenced by the solvent and the nature of X^−^ [45–46]. Anions like tetrafluoroborate, hexafluorophosphate, and trifluoromethanesulfate are commonly used in DAIS due to their good solubility and weak nucleophilicity. If the two aryl groups (Ar^1^ and Ar^2^) in DAIS are different, they are termed unsymmetric diaryliodonium salts **3**. The chemoselectivity of the product is primarily determined by the steric hindrance and electrophilicity of the aryl groups [17]. When the two aryl groups form a cyclic structure with a central iodine atom, they are referred to as cyclic diaryliodonium salts **4** (Figure 1). Cyclic DAISs are predominantly found in simple five to seven-membered cyclic compounds [47].

Arylation reactions using diaryliodonium salts can occur through four distinct mechanisms. First, the arylation can occur under metal-free conditions, involving the formation of a three-membered ring transition state through ligand coupling, leading to the formation of the Nu–Ar product and aryl iodide [21]. Second, the arylation can take place in the presence of a metal catalyst via oxidative addition, followed by reduction elimination [48–49]. Thirdly, it proceeds through a ligand-coupled arylation which involves a five-membered transition state to yield the respective arylation product [50–51]. Lastly, arylation can occur through single-electron transfer (SET), where a cation radical obtained from aromatic hydrocarbons with high electron density yields the desired arylated product [52]. In this review article, we will provide a comprehensive overview of arylation of carbon and heteroatom substrates via diaryliodonium salts in metal-free conditions. This review emphasizes the significance and potential of DIASs in contemporary organic chemistry.

## Review

### *C*-Arylation

Over the past decade, there has been a surge of interest in metal-catalyzed C-arylations utilizing diaryliodonium salts, marked by significant contributions, notably from research teams led by Sanford [53] and Gaunt [54]. The synthesis of carbon–carbon bonds through metal-free approaches serves as a valuable complement to transition-metal-catalyzed couplings. This is particularly significant as it circumvents the use of costly and hazardous metals and ligands which are commercially not available.

**Scheme 1:**
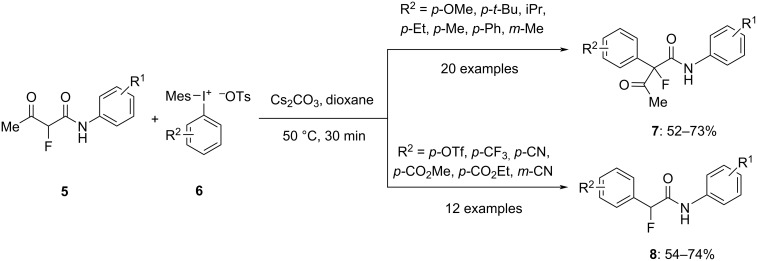
Αrylation of α-fluoroacetoacetamides **5** to α-aryl-α-fluoroacetoacetamides **7** and α-fluoroacetamides **8** using diaryliodonium salts **6**.

In order to obtain a variety of synthetically desirable tetrasubstituted α-aryl-α-fluoroacetoacetamides **7**, Zaheer et al. disclosed a straightforward, metal-free technique for the α-arylation of α-fluoroacetoacetamides **5** utilising unsymmetric DIAS **6**. Various α-fluoroacetoacetamides **5** with electronically different aliphatic, aryl ring, and heterocyclic substitutions were discovered to be easily arylated using this method. The products were obtained within 30 minutes in the presence of Cs_2_CO_3_ as shown in Scheme 1. The substrate scope exhibits that on using electron-deficient diaryliodonium salts as an arylating agent, α-fluoroacetamides **8** were obtained in moderate to good yields through a spontaneous arylation/deacylation cascade. The deacylation reaction is considered due to the presence of fluorine and a newly installed electron-deficient aryl group on α-carbon which increases electrophilicity of the α-carbon center [55].

**Scheme 2:**
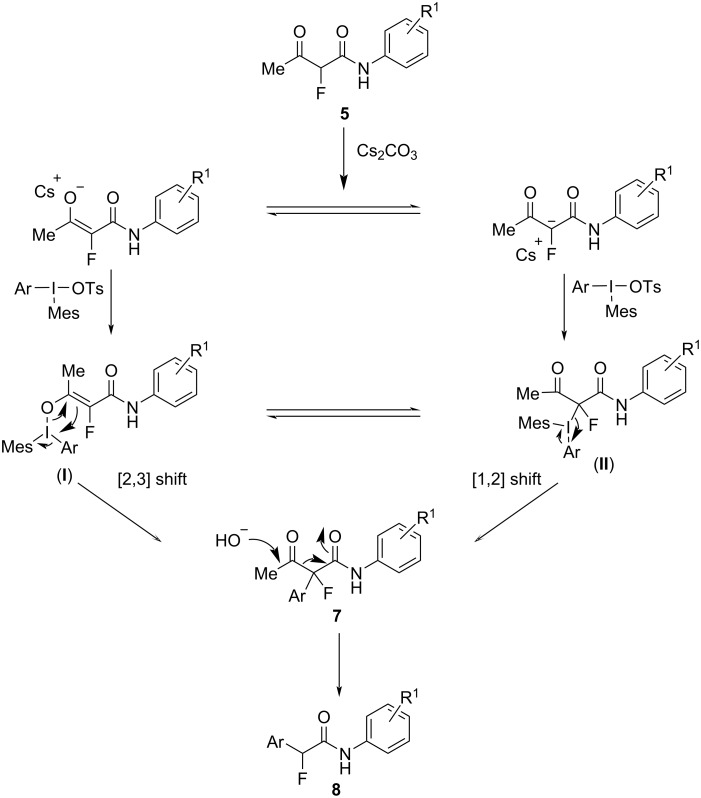
Proposed mechanism for the arylation of α-fluoroacetoacetamides **5** to α-aryl-α-fluoroacetoacetamides **7** and α-fluoroacetamides **8**.

The proposed reaction mechanism (Scheme 2) begins with the formation of one of two potential iodine intermediates, labeled as **I** or **II**. These intermediates arise upon binding of the enolate molecule to iodine either through a carbon–iodine or an oxygen–iodine bond. Both intermediates, **I** and **II**, are in rapid equilibrium with each other and further undergo two different types of reactions: [1,2]-ligand coupling and [2,3]-rearrangement (Scheme 2). Either of these reactions leads to the formation of the desired arylated α-aryl-α-fluoroacetoacetamides **7**. The tetrasubstituted fluorocarbon center becomes more electrophilic in the presence of an electron-deficient aryl (Ar) group. This increased electrophilicity facilitates a base-mediated deacylation reaction, resulting in arylfluoroacetamides **8** as final products.

**Scheme 3:**
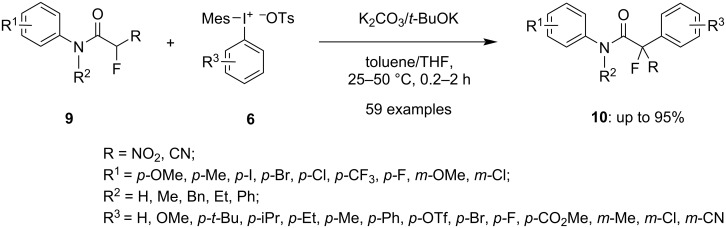
α-Arylation of α-nitro- and α-cyano derivatives of α-fluoroacetamides **9** employing unsymmetrical DAISs **6**.

Further, Zaheer and group developed an α-arylation of synthetically valuable α-fluoro-α-nitroacetamides (**9**, R = NO_2_, Scheme 3) under gentle conditions to form a quaternary benzylic fluorocarbon center. The protocol was found to be effective for the α-arylation of α-cyano-α-fluoroacetamides (**9**, R = CN), too. Aryl(mesityl)iodonium salts **6** (which are unsymmetrical diaryliodonium salts) were used as hypervalent iodine salts in both reactions. To achieve the C(sp^3^)-arylation of the α-nitro derivative of compounds **9** within 2 h to yield products **10**, K_2_CO_3_ as base and toluene as solvent were required (Scheme 3). On the other hand, for the α-arylation of the α-cyano derivative of compounds **9**, *t*-BuOK as base and THF as a solvent were useful to yield the products in a short reaction time (30 min). All the products with a wide range of electronically varied arenes were attained in good to excellent yields [56]. Additionally, the same reaction was further explored by using α-fluoro-α-nitrosulfonylmethanes as starting material under modified reaction conditions to yield the arylated α-fluoronitrosulfonylmethane [57]. Phenyl(mesityl)iodonium salt was employed to achieve the fluorinated products having a tetrasubstituted benzylic carbon center in good to excellent yields. The strategy was also used for the synthesis of α-arylated α-fluoro(arylsulfonyl)acetonitriles in good yield.

**Scheme 4:**
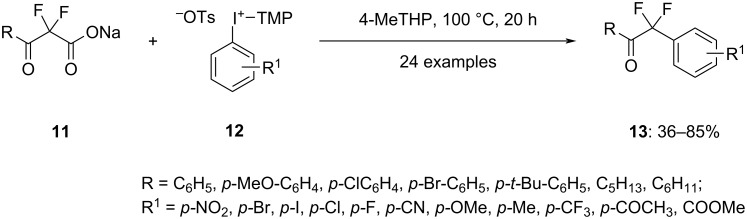
Synthesis of α,α-difluoroketones **13** by reacting α,α-difluoro-β-keto acid esters **11** with aryl(TMP)iodonium tosylates **12**.

In a recent study, Dohi et al. achieved the arylation along with decarboxylation of α,α-difluoro-β-keto acid esters **11** with the help of aryl(TMP)iodonium tosylates **12** in toluene at 100 °C to yield α,α-difluoroketones **13** in excellent yield (Scheme 4). The reaction proceeds via ligand exchange between the fluorinated carboxylate and the tosylate anion of the hypervalent iodine salt, subsequently leading to decarboxylative C–C coupling. Notably, this method achieves the incorporation of two fluorine atoms in the benzyl position without resorting to hazardous fluorination reagents, transition-metal catalysts, or organometallic compounds. The utility of this reaction is underscored by the successful conversion of various α,α-difluoromethyl ketone groups into corresponding esters, amides, and difluoromethyl groups [58].

**Scheme 5:**
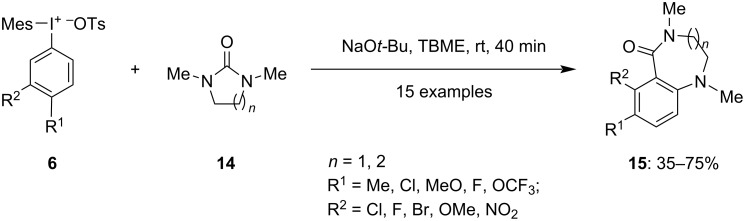
Coupling reaction of arynes generated by iodonium salts **6** and arynophiles **14** for the synthesis of tetrasubstituted arenes **15**.

In 2021, Nilova and colleagues outlined an approach for the synthesis of highly hindered 1,2,3,4-tetrasubstituted benzenoid rings **15** using arynes generated from **6** on reacting with arynophiles **14** (Scheme 5) [59]. The reaction is unique due to its ability to functionalize position 3, despite its greater steric hindrance compared to position 5. The process involves deprotonation at the 3-position of the aryl(Mes)iodonium salts, followed by exit of a leaving group from position 4, and then regioselective vicinal functionalization of the generated aryne. The method's compatibility with halide-substituted aryl compounds enhances its versatility and practicality. Moreover, the completion of reaction within a mere 40 minutes at room temperature underscores its efficiency and effectiveness as a synthetic approach.

**Scheme 6:**
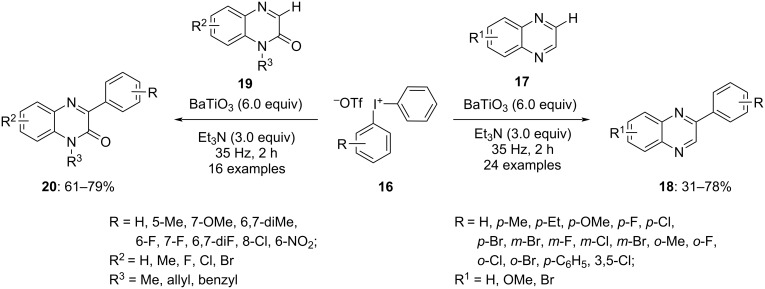
Metal-free arylation of quinoxalines **17** and quinoxalinones **19** with DAISs **16**.

Furthermore, a transition-metal-free arylation of quinoxalines **17** and quinoxalinones **19** via aryl radicals was discussed by Li and co-workers in 2022. In this report the aryl radicals were generated by planetary ball milling of diaryliodonium salts **16** at a frequency of 35 Hz in the presence of the piezoelectric material BaTiO_3_ (size < 4 µm)_._ The results were obtained within 2 h when triethylamine was used as a base (Scheme 6) [60]. Both, symmetrically and unsymmetrically substituted diaryliodonium salts were employed for the reaction and it was revealed that in unsymmetrical diaryliodonium salts the transfer of the aryl group with the relatively lower electron density and less steric hindrance was favoured. A range of electron-rich and electron-deficient substituents positioned *para* to the aryl ring in the diaryliodonium salts were found to be well tolerated in the reaction. Quinoxalines substituted at various positions resulted in the corresponding arylation products **18** in moderate yields. Under similar reaction conditions various substituted quinoxalinones yielded products **20** in moderate to good yields. The BaTiO_3_ used in the reaction could be easily recycled just by washing it with ethanol, retaining its catalytic activity for arylation up to three cycles without any compromise. Thus, this procedure could be considered economic as well as environment-friendly.

**Scheme 7:**
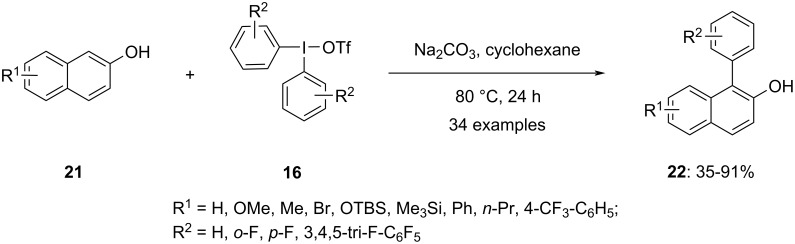
Transition-metal-free, C–C cross-coupling of 2-naphthols **21** to 1-arylnapthalen-2-ols **22** employing diaryliodonium salts **16** as the aryl source.

In 2019, Kalek and co-workers reported the regioselective C–H arylation of 2-naphthols **21** by using iodonium salts **16** as the source of the aryl group (Scheme 7) [61]. Through optimization, it was determined that the presence of Na_2_CO_3_ as base and cyclohexane as solvent facilitated the C–C cross-coupling reaction. The products were obtained in satisfactory yields using various diaryliodonium salts regardless of their differing counter-anions. A range of 2-naphthol substrates, including those bearing alkyl and aryl groups, halogens, trimethylsilyl, and protected hydroxy at positions 6 and 7, exhibited good tolerance. However, the reaction with 1-naphthol did not yield positive results. Notably, the efficiency of the cross-coupling reaction was observed to increase with the transfer of electron-poor aryl groups from the hypervalent iodine salt. Thus, electron-withdrawing substituents such as trifluoromethyl, *m*-chloro, and fluorine on the aryl group promoted efficient coupling.

**Scheme 8:**
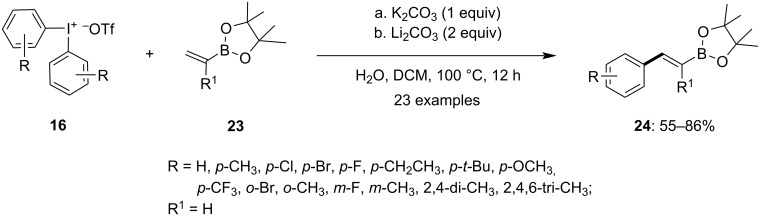
Arylation of vinyl pinacol boronates **23** to *trans*-arylvinylboronates **24** in presence of hypervalent iodine salt **16**.

Moreover, C–C bond formation was reported by Chen and colleagues in 2020 via the arylation of vinyl pinacol boronates **23** by using diaryliodonium salts **16** to yield *trans*-arylvinylboronates **24** in the absence of a metal catalyst [62]. The optimized reaction conditions involve the reaction of substituted diaryliodonium salts **16** with different substituted vinyl pinacol boronates **23** in dichloromethane as solvent at 100 °C in a sealed tube in the presence of a wet inorganic base (Scheme 8). Both K_2_CO_3_ and Li_2_CO_3_ were found to be compatible with the reaction, and it was observed that no product was obtained in the absence of a base. Additionally, the presence of 40 equivalents of water proved to be crucial for the reaction, as altering the amount of water significantly impacted the product yield, indicating the importance of water in the reaction mechanism. A diverse range of functionalized diaryliodonium salts, including di- and trisubstituted ones, were well tolerated in the reaction, providing products **24** with good stereoselectivity in moderate to good yields. Moreover, the reaction was successfully conducted with various substituted vinyl pinacol boronates and di(4-tolyl)iodonium triflate, resulting in moderate to good yields of the corresponding products. The vinyl boronates obtained from the aforementioned reaction were subsequently subjected to a Suzuki coupling with the remaining aryl iodides obtained from **16** in the presence of a palladium catalyst. This step facilitated the formation of functionalized olefins, showcasing an efficient utilization of aryliodonium salts in the process.

**Scheme 9:**
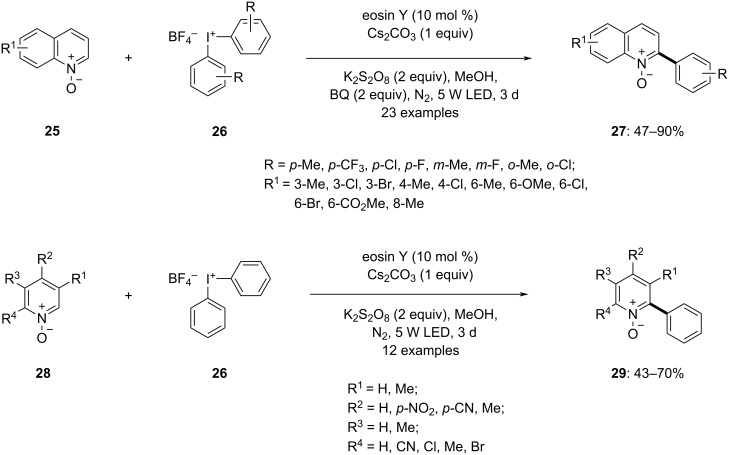
Light-induced selective arylation at C2 of quinoline *N*-oxides **25** and pyridine *N*-oxides **28** in the presence of **26**.

Later in the same year, Song and colleagues reported a protocol for the efficient synthesis of 2-aryl-substituted quinolines **27** and pyridine *N*-oxides **29** [63]. This reaction involved the selective arylation at the C2 position of quinoline *N*-oxides and pyridine *N*-oxides, utilizing hypervalent iodine salts as the arylation reagents. The reaction was facilitated by visible light in conjunction with a photocatalyst. The absence of either the photocatalyst or light resulted in only trace amounts of the product, underscoring their essential roles in product formation. Optimized conditions comprised the reaction of the quinoline *N*-oxides **25** (1 equiv) with diaryliodonium tetrafluoroborates **26** (2 equiv) as the arylating agent, 1,4-benzoquinone (BQ) as an additive (2 equiv), the photocatalyst eosin Y (10 mol %), and Cs_2_CO_3_ (1 equiv) as the base in methanol under a nitrogen atmosphere, with 5 W LEDs irradiation for 3 days (Scheme 9). Various substituted quinone *N*-oxides yielded the corresponding products in satisfactory to moderate yields. Notably, electron-withdrawing groups generated higher yields compared to electron-donating groups. The reaction exhibited high selectivity, with substitution at the C3 position not impeding the reaction. Different substituted diaryliodonium tetrafluoroborates were also investigated, yielding good product yields. The above protocol for the arylation of pyridine *N*-oxides **28**, resulted in corresponding products in moderate to good yields. The reaction conditions remained consistent, except K_2_S_2_O_8_ was found to be a superior additive compared to BQ. The reaction exhibited good tolerance even towards strong electron-withdrawing groups.

**Scheme 10:**
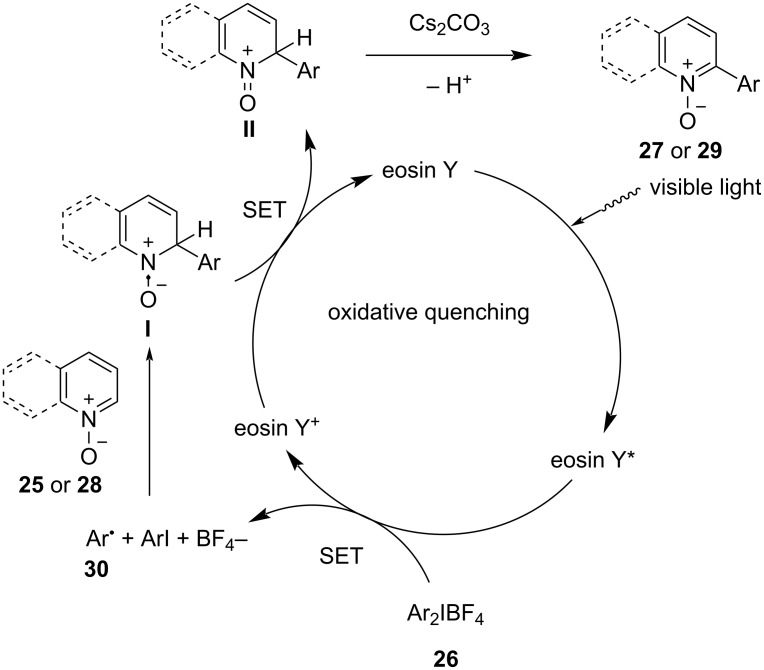
Plaussible mechanism for the light-induced selective arylation of *N*-heterobiaryls.

A control experiment was conducted to investigate the reaction mechanism by adding 2 equivalents of TEMPO to the reaction mixture. The absence of the desired product indicated the involvement of a radical pathway in the process. The proposed reaction mechanism begins with the activation of eosin Y by visible light from 5 W blue LEDs, transitioning it to its excited state, eosin Y*. This excited state further undergoes oxidation via a single-electron-transfer (SET) reaction with Ar_2_IBF_4_ **26**, producing eosin Y^+^ and a phenyl radical **30** (Scheme 10). The radical intermediate **30** selectively binds to the C2 position of either quinoline or pyridine *N*-oxide, forming intermediate **I**. Furthermore, intermediate **I** subsequently undergoes another SET reaction, resulting in intermediate **II** and the regeneration of the photocatalyst. Intermediate **II** undergoes deprotonation, facilitated by the presence of Cs_2_CO_3_ as base, to yield the final products **27** or **29**. Additives like BQ likely assist in the deprotonation of intermediate **II** to produce final products **27**, while K_2_S_2_O_8_ aids in the oxidation of the photocatalyst in the case of pyridine *N*-oxide.

**Scheme 11:**
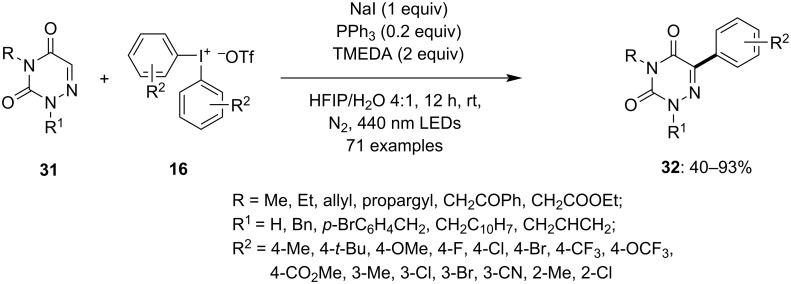
Photoinduced arylation of heterocycles **31** with the help of diaryliodonium salts **16** activated through donor–acceptor complex formation.

In another photoinduced reaction procedure, Murarka et al. reported the formation of aryl radicals from a tetrameric electron donor–acceptor (EDA) complex. The complex is formed of triphenylphosphine, sodium iodide and *N*,*N*,*N*,*N*-tetramethylethylenediamine (TMEDA) with diaryliodonium reagents (DAIRs) [64]. This activates DAIRs **16** to generate an aryl radical which is utilized in the C–H arylation of various heterocycles **31** to yield the corresponding heteroaryl–aryl compounds **32** in moderate to good yield. The use of blue LEDs (456 nm), nitrogen atmosphere, and HFIP/H_2_O 4:1 solvent mixture improved the yield of the product by up to 90%. Various substituted azauracils were used to study the reaction and it was observed that different substituted N2/N4 azauracils were easily converted to the corresponding products in good to excellent yield. Furthermore, diverse DAIRs were subjected to the reaction which again provided the desired products **32** in moderate to excellent yield (Scheme 11). The study suggests that the unsymmetrical DAIRs transfer the aryl ring which is less sterically hindered. The reaction conditions enabled to furnish results with various aromatic and nonaromatic heterocycles and *N*-heterocyles. The reaction was able to facilitate late-stage diversification of drug molecules such as nimesulide and gemfibrozil to corresponding products. The radical path was considered for the reaction mechanism as on adding TEMPO as radical scavenger the radical trapping adduct was detected by HRMS.

**Scheme 12:**
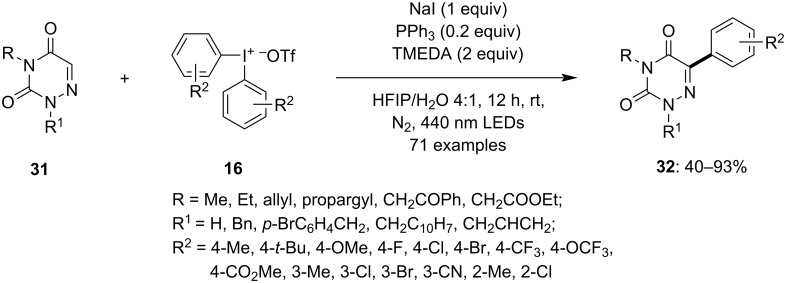
Arylation of MBH acetates **33** with DIPEA and DAIRs **16**.

Simultaneously with the above work, Murarka and co-workers also reported an organophotoredox-catalyzed stereoselective allylic arylation method for Morita–Baylis–Hillman (MBH) acetates using a variety of diaryliodonium triflates [65]. The reaction was carried out with MBH acetate **33** and diphenyliodonium triflate **16** in the presence of different photocatalysts and bases. Methylene blue trihydrate (MB·3H_2_O) was identified as a highly active photosensitizer and DIPEA was effective as a base (Scheme 12). The reaction yielded the *E*-isomers in a solvent mixture of methanol/water 5:1 under blue LED light (467 nm) irradiation in good yields. Various DAIRs substituted at different positions were found to be suitable for the reaction giving the respective products in moderate to good yield. Additionally, unsymmetrical DAIRs showed a preference for transferring an electron-poor and sterically less hindered aryl ring. The scope of MBH acetates was further explored, demonstrating compatibility with a wide range of substituted aromatic ethyl acrylates and various substituents on the aromatic ring. Additionally, a variety of substituted esters, ketones, and nitriles were found to be compatible with the reaction.

**Scheme 13:**
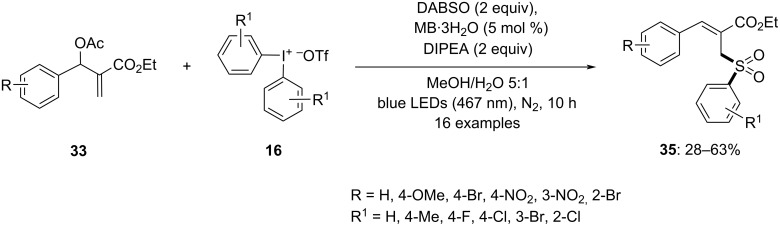
Aryl sulfonylation of MBH acetates **33** with DABSO and diphenyliodonium triflates **16**.

Additionally, the same approach was used for the aryl sulfonylation. Trisubstituted allylic sulfones **35** were synthesized by reacting MBH acetate **33** and diphenyliodonium triflates **16** using an optimal sulfur dioxide surrogate, 1,4-diazabicyclo[2.2.2]octane bis(sulfur dioxide) (DABSO, Scheme 13). The *Z*-isomer of the desired products was obtained by optimizing the reaction conditions. The involvement of radicals in both the arylation and aryl sulfonylation was confirmed as no product was found when carrying out the reaction in the presence of radical scavengers. Stern–Volmer studies indicated a significant fluorescence emission quenching of methylene blue by DIPEA, suggesting a reductive quenching of methylene blue during the reaction. Experiments involving variations in light exposure and quantum yield established the need for continuous irradiation and eliminated the possibility of a radical chain mechanism for the reaction.

**Scheme 14:**
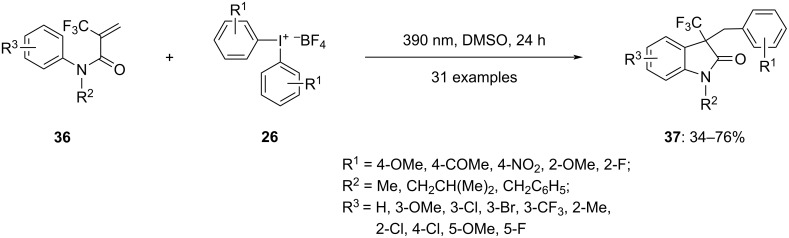
Synthesis of oxindoles **37** from *N*-arylacrylamides **36** and diaryliodonium salts **26**.

Recently in 2023, Yadav and colleagues demonstrated that diaryliodonium salts are effective for arylating and cyclizing trifluoromethylated acrylamides **36** under environmentally friendly conditions [66]. When the reaction mixture of acrylamides **36** and diaryliodonium tetrafluoroborates **26** dissolved in water was irradiated with light of 390 nm wavelength, the desired oxindole products **37** were obtained in good yields (Scheme 14). The reaction was notably more successful with CF_3_-acrylamide than with CH_3_-acrylamide, likely because the former dissolves more readily in water. The reaction tolerated diverse substitutions at different positions on the aryl groups of acrylamides **36** and diaryliodonium salts **26**. Both electron-withdrawing and electron-donating substituents at the *para*-position of the *N*-arylacrylamide led to good yields. In cases of *meta*-substitution, a mixture of C6 and C4-substituted oxindole products were obtained, whereas *ortho-*substitution resulted in the desired oxindoles in moderate yields. Nitrogen substitution was also found to be tolerable in the reaction. Interestingly, substituting the *para*-position of the aryliodonium salts with an electron-donating group resulted in a moderate product yield, while electron-withdrawing groups led to decreased yields of the desired product.

### *N*-Arylation

**Scheme 15:**
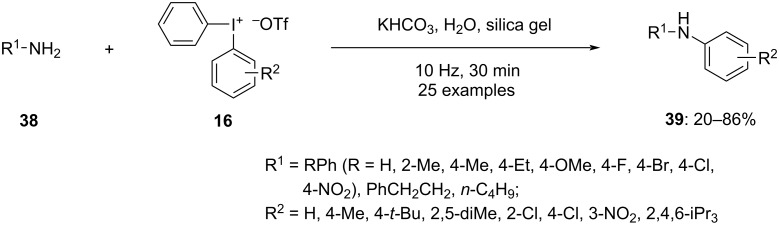
Mechanically induced *N*-arylation of amines **38** using diaryliodonium salts **16**.

Nitrogen-containing heteroaromatic compounds serve as crucial scaffolds in pharmaceuticals. Therefore, the synthesis of *N*-substituted heteroaromatic derivatives under mild and environmentally friendly conditions is highly valued. The use of electrophilic diaryliodonium salts for nitrogen arylation has been investigated. Working on this Li and Jiang, created an effective ball-milling process for the *N*-arylation of amines **38** with the assistance of diaryliodonium salts **16** (Scheme 15) [67]. The arylation of an amino group or nitrogen heterocycle occurred effectively when the reaction is performed under solvent-free conditions or when a minimal quantity of water was used as a solvent in the presence of a base. The ball-milling method efficiently simplified the reaction process because, in contrast to typical solution methods, it may realize product formation without being affected by the solubility of the substrate and other additives. An efficient conversion was detected when the substrate contains electron-rich functionalities. In contrast, the yield dropped notably when the amines were substituted with electron-deficient functionalities. In addition, asymmetric diaryliodonium salts were examined, and the transfer of the aryl group with a less hindered portion is observed. The mechanism revealed the reaction undergoes the homolytic cleavage of the diaryliodonium salt to produce an iodoaryl radical cation, which further reacts with the amine to acquire the corresponding diaryl amines. Moreover, a similar reaction tried with a copper catalyst afforded nearly better results for the arylated products.

**Scheme 16:**
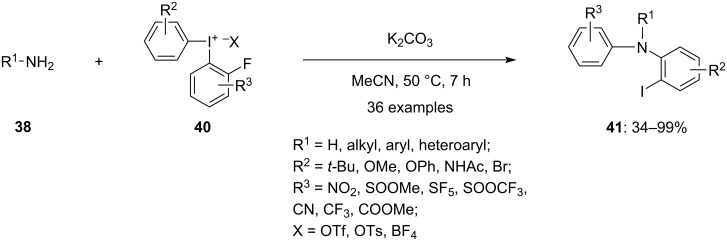
*o*-Fluorinated diaryliodonium salts **40**-mediated diarylation of amines **38**.

In 2022, Linde et al., demonstrated a conventional approach for achieving arylations of nitrogen- and oxygen nucleophiles via S_N_Ar reaction, using *o*-fluorinated diaryliodonium salts **40**, which enabled access to a greater range of compounds (Scheme 16) [68]. The novel iodine(III) intermediate was generated through nucleophilic substitution of a heteroatom nucleophile, which initiated the reaction. A subsequent aryl migration from the iodine to the heteroatom resulted in the formation of the arylated nucleophile. In addition to accepting a wide variety of protective and functional groups, the method creates products with an iodine substituent that is easily accessible for product derivatization. Moreover, it is a convenient methodology for both *N*-arylation and *O*-arylation. The arylation of amines **38** was achieved in acetonitrile as solvent, whereas the arylation of ammonia was achieved by using ethyl acetate as solvent along with potassium carbonate as a base. Likewise, water was arylated using cesium carbonate as a base. The *N*-arylation reactions were performed under strict anhydrous conditions.

**Scheme 17:**
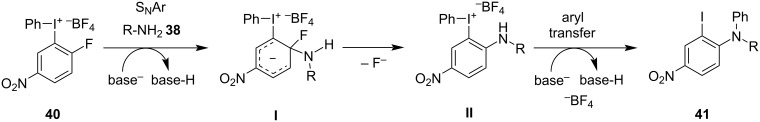
Proposed mechanism for the diarylation of amines **38** using *o*-fluorinated diaryliodonium salts **40**.

The reported mechanistic hypothesis (Scheme 17) suggests that the reaction initiates with an S_N_Ar at the *ortho*-carbon, forming a Meisenheimer complex **I** and a novel iodine(III) intermediate **II**. This type of reactivity is unprecedented, as past reactions between nucleophiles and diaryliodonium salts usually lead to a reduction of iodine(III) to iodine(I). Intermediate **II** then undergoes an intramolecular aryl migration, yielding the diarylated products **41**, analogous to the known iodoniumphenolate reactions that produce diaryl ethers.

**Scheme 18:**
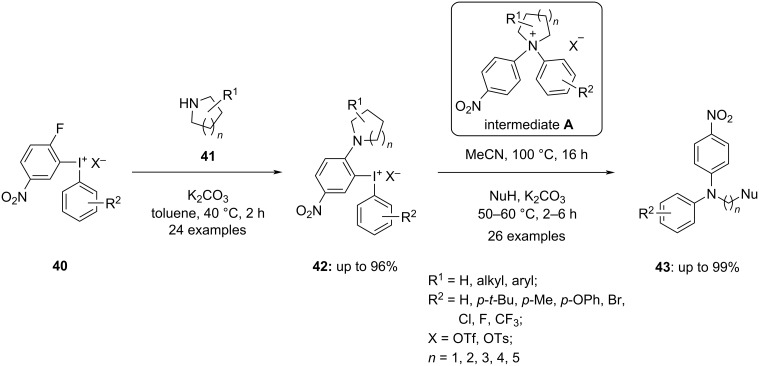
Ring-opening difunctionalization of aliphatic cyclic amines **41**.

The same research group meliorated the *N*-arylation of aliphatic cyclic amines with the same fluorinated diaryliodonium salts **40** (Scheme 18) [69], which however, does not produce the diarylated compounds. The intramolecular aryl migration from the iodine to the nitrogen leads to a quaternary ammonium ion intermediate **A**. Consequently, a nucleophilic ring opening of cyclic amine in **42** occurred via cleavage of the strong C–N bond. The ring opening incorporated nitrogen, oxygen, sulfur, carbon, and halogen-containing nucleophiles and their derivatives. The substrate scope was examined with numerous aryl groups on iodonium salts **40** and the progress of aryl migration happens fruitfully by considering electronic factors like steric hindrance. The ring opening proceeded smoothly when nucleophiles with higher nucleophilicity are used yielding up to 99% of the desired products **43**.

**Scheme 19:**
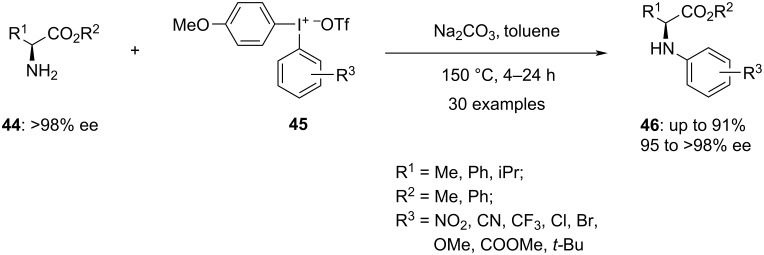
*N*-Arylation of amino acid esters **44** using hypervalent iodonium salts **45**.

The *N*-arylation of amino acid esters **44** was established with the utility of substituted phenyl(anisyl)iodonium triflate salts **45** (Scheme 19) [70]. According to the screening studies, a mixture of enantiomers was obtained, with one enantiomer predominating (95 to >98% ee) under the optimized conditions. The phenyl with electron-deficient groups is well tolerated and produces outstanding yields. Moreover, the phenyl with a bulky substituent also participated with the high yield of corresponding product. Further, the reaction with iodonium salts with electron-donating substituents in the aryl ring required an extension of the reaction duration to 24 hours. Furthermore, the scope of substrates was investigated, with a focus on the benzyl ester generated rather than the corresponding methyl esters. The reaction was also performed with a 6-membered cyclic diaryliodonium salt, which proceeded successfully and produced the respective iodo-containing arylated product in 59% yield with 76% ee after 24 h. Using the same reaction conditions, the arylation of tyrosine methyl ester was also performed. The resulting compound was arylated at both *O*- and *N*-positions. The investigations were continued with the unsymmetric anisyl salts, and the results showed high chemoselectivity for *N*-arylation. Iodonium salts containing the anisyl auxiliary enhanced the arylation yields.

**Scheme 20:**
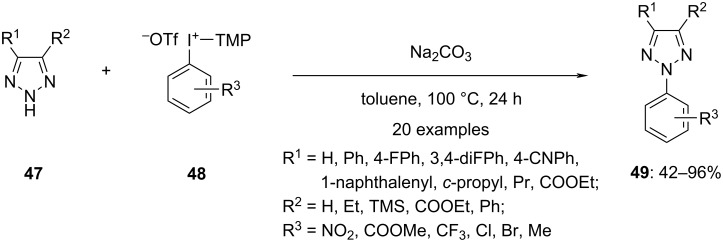
Regioselective *N*-arylation of triazole derivatives **47** by hypervalent iodonium salts **48**.

**Scheme 21:**
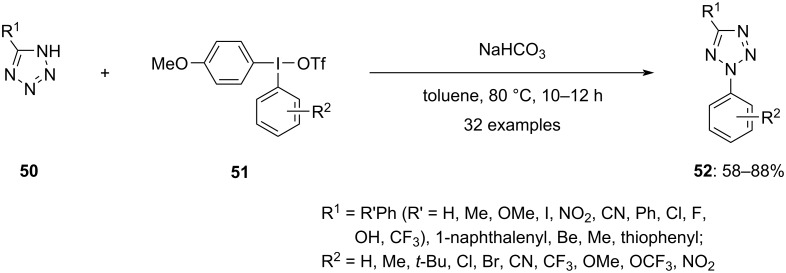
Regioselective *N*-arylation of tetrazole derivatives **50** by hypervalent iodonium salt **51**.

Hypervalent iodonium salts are also useful to achieve the *N*-arylation of azoles. Prakash and co-workers applied iodonium salts **48** in the presence of a base to obtain regioselectively the N2-arylated products of 1,2,3-triazoles via ligand exchange followed by reductive elimination in exceptional yields (Scheme 20) [71]. Although screening studies indicated the possibility of achieving the *N*-arylation at both, the N1- and N2-positions of the triazoles, N2-arylation was predominantly observed. It was incredible to achieve splendid regioselectivity without the usage of directing groups and any metal catalyst. Also, the electronic nature of a substituent at the C4 position of the starting triazole did not negatively impact the regioselectivity. Further, C4 and C5 disubstituted triazoles also produced the N2-arylated product. Remarkably, this is the only approach providing regioselective access to N2-arylated products along with high yields. The synthetic route could also be applied for the synthesis of N2-arylbenzotriazoles which are promising scaffolds for pharmaceuticals. Subsequently, the impact of the aryl group present in the diaryl iodonium salts on the reaction efficiency and selectivity was explored. A high selectivity was found for electron-withdrawing moieties, resulting in high yields of the N2-substituted products. Also, tetrazole **50** was arylated using the same hypervalent iodonium salts as a follow-up, but less than 14% of the targeted product were obtained. However, the yield of products **52** could be improved up to 66% by using iodonium salt **51** having the TMP group substituted with anisyl (Scheme 21) [72].

**Scheme 22:**
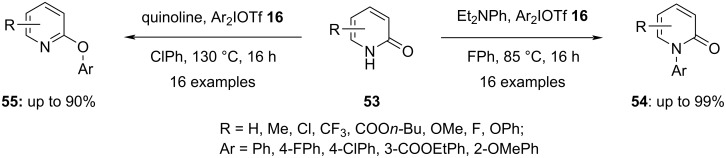
Selective arylation at nitrogen and oxygen of pyridin-2-ones **53** by iodonium salts **16** depending on the base and solvent used.

Switching the base in the arylation process can influence the chemoselectivity of the reaction as was reported by Onomura and group. They observed that the reaction of 2-pyridones **53** gave either the *N*- (**54**) or *O*-arylated product **55** as major component depending on the base used. Ultimately, the study progressed to optimized conditions leading selectively to either product. Briefly, when diethylaniline was used as a base in fluorobenzene, the *N*-arylated compounds **54** were produced. On the other hand, the *O*-arylated compounds **55** emerged as major product when quinoline was used as the replacement base and chlorobenzene as the solvent in the reaction (Scheme 22). The effect of the substituent on the aryl group in the hypervalent iodonium salt, was investigated, and the ratio of aryl group migration was found to depend on steric and anti-*ortho* effects [73].

**Scheme 23:**
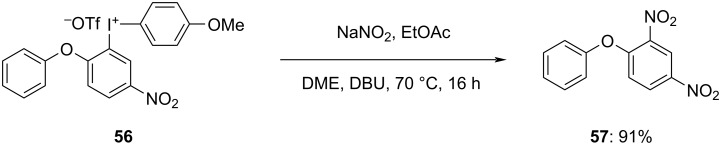
*N*-Arylation using oxygen-bridged acyclic diaryliodonium salt **56**.

Oxygen-bridged cyclic diaryliodonium salts are novel arylating reagents recently developed by Linde et al. and were utilized to arylate carbon and heteroatoms. However, the reactivity of these cyclic salts was found to be limited, which prompted the authors to synthesize the corresponding acyclic iodonium salt **56** to increase the reactivity. It was subsequently employed in various arylation processes of several substrates. Remarkably, as much as 98% of the targeted *N*-arylated compound **57** was obtained by treating hypervalent iodine salt **56** with NaNO_2_ in the presence of DBU as the base (Scheme 23). This salt was also studied for the arylation of sulfur, oxygen, and carbon giving good yields of corresponding products [74].

### *O*-Arylation

Arylation of oxygen is a significant chemical reaction that results in the formation of diaryl ethers. Diaryl ethers are important structural motifs in pharmaceuticals and agrochemicals due to their diverse biological activities. Since the 1950s, one of the most used methods for the synthesis of diaryl ethers involves the reaction of phenol with diaryliodonium salts. Recent advancements in this field have focused on the development of practically simple and scalable methods for the arylation of oxygen using diaryliodonium salts. By modifying the counter anions attached to the iodonium ion, the stability and reactivity of new symmetrical and unsymmetrical diaryliodonium salts could be improved which were subsequently used to synthesize new oxygen arylated products.

**Scheme 24:**
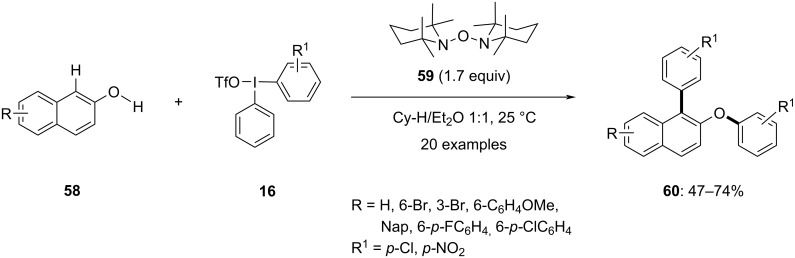
The successive C(sp^2^)–C(sp^2^)/O–C(sp^2^) bond formation of naphthols **58**.

Solorio-Alvarado and co-workers introduced a one-pot double arylation of naphthols through the consecutive C–C/O–C bond formation in the presence of hypervalent iodine salts **16** as the aryl donor (Scheme 24) [75]. The reaction worked very well at room temperature under base-free conditions. In this one-pot synthesis of double arylation of naphthols **58**, a novel radical precursor, [1,1´-oxybis(2,2,6,6-tetramethylpiperidine)] (**59**), was employed. This precursor undergoes spontaneous homolytic fragmentation in solution, producing tetramethylpiperidinyl radical and the TEMPO radical. The tetramethylpiperidinyl radical interacts with 2-naphthol derivatives **58**, leading to the generation of an oxygen-centered radical through hydrogen atom transfer, which resonates with its respective carbon-centered radical. Subsequently, these O- and C-centered naphthyl radicals selectively react with hypervalent iodine salts **16** at their more electron-poor hypervalent bond, preferentially transferring the more electron-deficient aryl group to yield the double arylated products **60** in moderate to good yields.

**Scheme 25:**
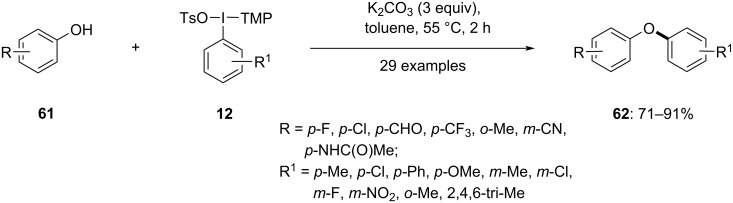
Synthesis of diarylethers **62** via in situ generation of hypervalent iodine salts.

A synthetic protocol for diaryl ethers via an in situ generation of a hypervalent iodine salt was introduced by Stuart and co-workers in 2020. To study the scope of the reaction first various substituted aryl(TMP)iodonium salts **12** were reacted with different substituted phenols **61** in the presence of K_2_CO_3_ at 55 °C to yield the corresponding products **62** in good to excellent yield (Scheme 25) [76]. It was observed that both electronic as well as steric effects on the aryl electrophile and phenol nucleophile were well tolerated. Further, this study was used for the one-pot synthesis of diaryl ethers **62**, starting with aryl iodides and phenols **61**. In this metal-free reaction, aryl(TMP)iodonium salts **12** were prepared in situ from aryl iodides via treatment with *m*-CPBA, TsOH, and TMB at 55 °C in acetonitrile, which subsequently react with the substituted phenols **61** to produce the *O*-arylated products **62**. Acetonitrile was identified as a suitable solvent for this reaction, resulting in moderate to good yields of the products. The three main steps in the reaction were oxidation of the aryl iodide, addition of the TMP auxiliary, and C–O coupling reaction.

**Scheme 26:**
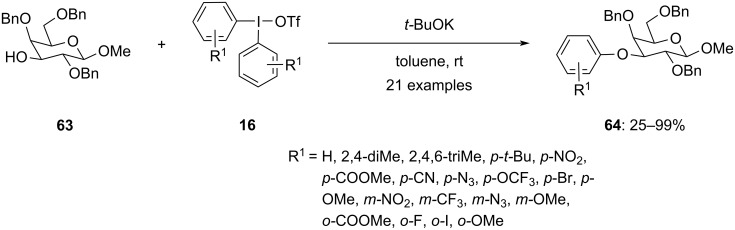
*O*-Arylated galactosides **64** by reacting protected galactosides **63** with hypervalent iodine salts **16** in the presence of base.

Olofsson et al. worked towards the synthesis of O3-arylated galactosides **64** by reacting benzyl-protected galactoside **63** with diphenyliodonium triflates **16** at room temperature in the presence of potassium *tert-*butoxide as the base (Scheme 26) [77]. This transition-metal-free approach simplifies the synthesis process. Electron-pushing and -pulling groups at the *para* and *meta*-position of the aryl group were compatible with the reaction leading to moderate to good yields of products. In contrast, products with substitutions at the *ortho* position were obtained only after heating the reaction mixture to 60 °C. These compounds were further assessed as inhibitors of galectin-9 and were found to exhibit selectivity and potency against galectin-9.

**Scheme 27:**
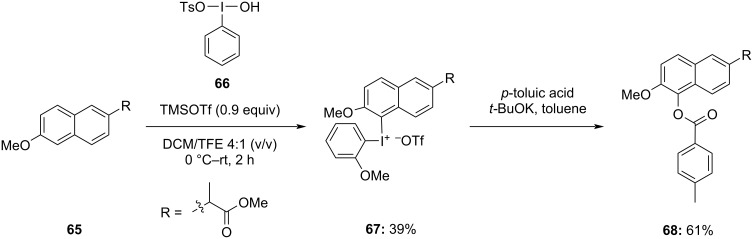
Esterification of naproxen methyl ester **65** via formation and reaction of naproxen-containing diaryliodonium salt **67** with *p*-toluic acid.

In 2021, Chen and colleagues developed a method to synthesize naproxen-containing diaryliodonium salts **67** using naproxen methyl ester **65** and ArI(OH)OTs **66**, activated by trimethylsilyl trifluoromethanesulfonate (TMSOTf). This synthesis was conducted in a mixture of 2,2,2-trifluoroethanol (TFE) and dichloromethane [78]. The synthesized naproxen-containing diaryliodonium salt **67** was further used to modify the aromatic ring of naproxen methyl ester **68** (Scheme 27). Various functionalization reactions which include arylation, iodination, alkynylation, thiophenolation, amination, and esterification, were carried out. Among these reactions, esterification was achieved in moderate yield under metal-free conditions by reacting the synthesized naproxen methyl ester (2-methoxyphenyl)iodonium trifluoromethanesulfonate with *p*-toluic acid in the presence of *t*-BuOH.

**Scheme 28:**
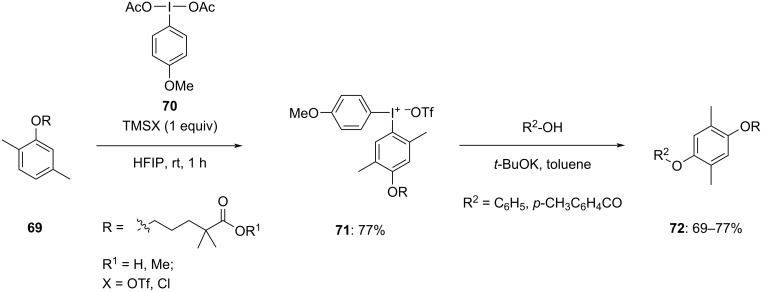
Etherification and esterification products **72** through gemfibrozil methyl ester-derived diaryliodonium salts **71**.

Moreover, later in 2022 Chen et al. modified the aromatic ring of gemfibrozil (**69**) and its methyl ester using gemfibrozil-derived diaryliodonium salts **72** synthesized by the aforementioned procedure [79]. On reacting gemfibrozil **69** in the presence of bis(4-methoxyphenyl)iodonium diacetate (**70**) or ArI(OH)OTs highly regioselective gemfibrozil methyl ester derived iodonium salts **71** were obtained in moderate to good yield. These salts were then used for various modifications like alkynylation, arylation, esterification, etherification, fluorination, and iodination of the gemfibrozil aromatic ring by reacting them with the corresponding nucleophiles. Notably, reactions with phenol, thiophenol and benzoic acid using salts **71** in the presence of *t*-BuOK led to the corresponding products **72** with 61%, 69%, and 77% yields, respectively (Scheme 28). These reactions occurred under mild conditions and without any need of transition-metal catalysts.

**Scheme 29:**
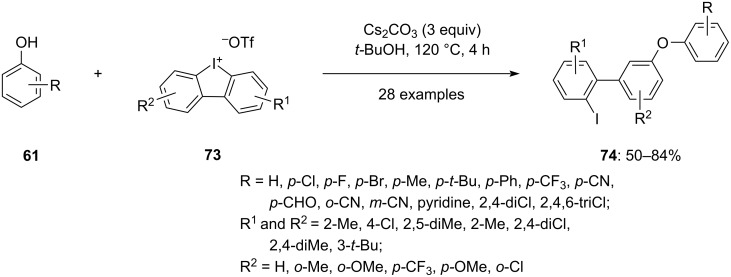
Synthesis of iodine containing *meta*-substituted biaryl ethers **74** by reacting phenols **61** and cyclic diaryliodonium salts **73**.

In 2023, Wu and colleagues successfully synthesized a range of *meta*-substituted biaryl ethers. The reaction involves phenols **61** and cyclic diaryliodonium salts **73**, dissolved in *tert*-butyl alcohol, in the presence of the base Cs_2_CO_3_ yielding iodine-containing *meta*-functionalized biaryl ethers **74** (Scheme 29) [80]. Notably, the reaction occurs under transition-metal-free conditions, making it environmentally friendly. The team explored the substrate scope by introducing various substitutions on both phenols **61** and diaryliodonium salts **73**. Remarkably, the method exhibited high regioselectivity, with the substitution at the *meta* position being observed with up to 99% selectivity in comparison to the *ortho* position. Specifically, only electron-withdrawing groups like OMe or CF_3_ when substituted at the *m*-position relative to the iodine center give the *ortho*-substituted products in good yield. Additionally, *ortho*-disubstituted diaryliodonium salts also led to the formation of *ortho*-substituted biaryl ethers in good yield with 99% selectivity and the reason given was the high torsional strain caused by the *ortho*-disubstitution on the diaryliodonium salts.

**Scheme 30:**
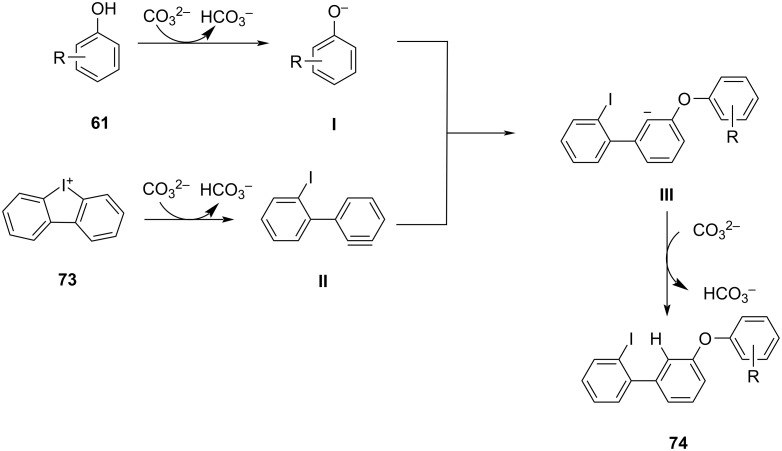
Plausible mechanism for the synthesis of *meta*-functionalized biaryl ethers **74**.

The reaction mechanism involves the deprotonation of the phenol **61** and diaryliodonium salts **73** via base under high temperature to get phenolate **I** and benzyne intermediate **II**, respectively. The phenolate nucleophile reacts with the benzyne intermediate to create a C–O bond, leading to the formation of the carbanion intermediate **III**. Lastly, this intermediate is protonated by bicarbonate to yield the final product **74** (Scheme 30).

**Scheme 31:**
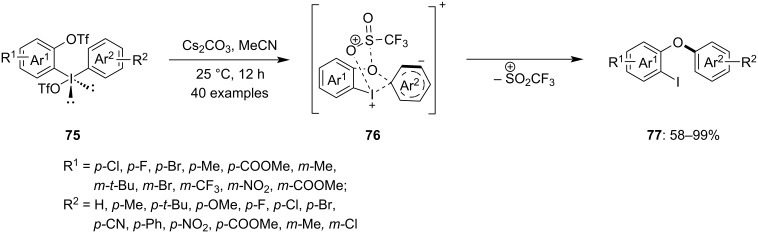
Intramolecular aryl migration of trifluoromethane sulfonate-substituted diaryliodonium salts **75**.

Furthermore, Wang and his team introduced a novel method to synthesize *ortho*-iodo diaryl ethers **77** using intramolecular aryl migration in trifluoromethanesulfonate-substituted diaryliodonium salts **76** [81]. This reaction occurs under basic conditions at temperatures ranging from room temperature to 50 °C and is completed within 12 hours (Scheme 31). The process is atom-efficient as no aryl residue is wasted as a byproduct. Various functional groups on both aromatic rings were investigated, and based on the obtained yields of the products it was concluded that the reaction is compatible with electron-donating, electron-withdrawing, and electron-neutral substitutions. Mechanistic studies, including a cross-over reaction, indicated that the aryl migration is intramolecular. In the presence of a base, the triflate anion is extracted, forming a cationic trifluoromethanesulfonyl group as an intermediate. Thus, the products are believed to form via a sulfonyl-directed nucleophilic aromatic substitution pathway. Finally, the products are obtained through the dissociation of the SO_2_CF_3_ leaving group from the intermediate.

**Scheme 32:**
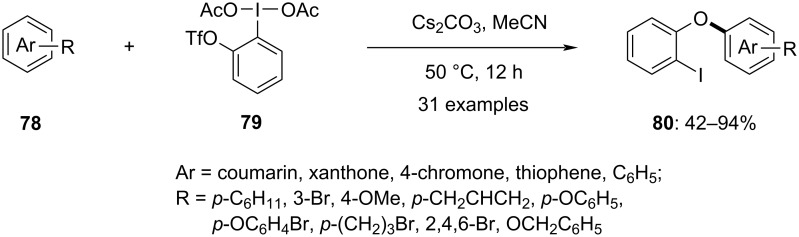
Synthesis of diaryl ethers **80** via site-selective aryl migration.

For the late-stage substitution of coumarins, Han and colleagues developed a method, using hypervalent iodine reagents. At 50 °C in the presence of a base, coumarin-based aryliodonium salts are produced. These salts undergo an intramolecular aryl rearrangement to form C(sp^2^)–O bonds without the need of metal catalysts [82]. In 2023, this approach was expanded to synthesize complex functionalized aromatic ring diaryliodonium salts [83]. Various aromatic rings, including multisubstituted arenes, conjugated arenes, oxygen and nitrogen heterocycles **78** were utilized to prepare these salts using *ortho*-triflate-substituted iodobenzene acetate **79**. All synthesized diaryliodonium salts underwent successful aryl migrations, yielding the expected products **80** efficiently (Scheme 32). The advantages of this method include late-stage site-selective *O*-arylation, transition metal-free conditions, and the presence of a C–I bond in the product, allowing for further functionalization through various coupling reactions, making the reaction method highly attractive.

**Scheme 33:**
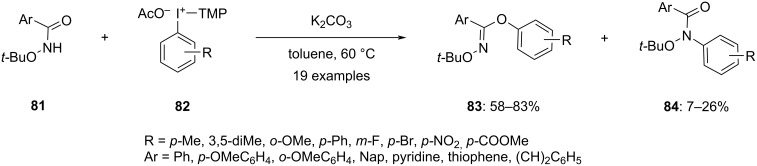
Synthesis of *O*-arylated *N*-alkoxybenzamides **83** using aryl(trimethoxyphenyl)iodonium salts **82**.

In 2022, Dohi and colleagues emphasized the high reactivity of TMP-iodonium acetates in the *O*-arylation of phenol derivatives [84]. The study revealed that the blend of the TMP ligand and the acetate anion in iodonium salts **82** synergistically increased electrophilic reactivity. The *ortho*-methoxy groups of TMP boost the basicity of the acetate anion by coordinating with the iodine(III) center, which facilitates the deprotonation of phenols. This method was well-suited for various functional groups, yielding diaryl ethers with significantly improved yields compared to other diaryliodonium salt reactions. Furthermore, the same research group extended the use of aryl(trimethoxyphenyl)iodonium salts for *O*-arylation of *N*-alkoxybenzamides **81** in absence of metal catalsyt at low temperatures [85]. The reaction resulted in the two products *O*-arylated **83** and *N*-arylated **84** amides (Scheme 33). By reacting various substituted diaryliodonium salts and the amide it was concluded that the chemoselectivity between *O*- and *N*-arylation could be controlled by adjusting the steric and/or electronic properties of the diaryliodonium salt and the amide. This approach was helpful in the formation of *O*-arylimidates previously unattainable using metal-catalyzed methods.

### *S*-Arylation

**Scheme 34:**
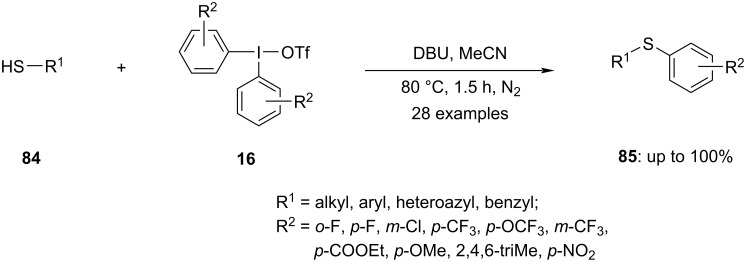
Synthesis of aryl sulfides **85** from thiols **84** using diaryliodonium salts **16** in basic conditions.

The aryl sulfide moiety is widely present in biologically active compounds and natural products. Consequently, the synthesis of aryl sulfides has drawn increasing attention. Diaryliodonium salts have been reported to be utilized to arylate thiols in a number of publications in recent years [44,86–87]. In 2022, Sarkar et al. demonstrated a synthesis of aryl sulfides **85** from thiols **84** using diaryliodonium salts **16** in basic conditions (Scheme 34). A multitude of thiols and diaryliodonium salts was examined in the reaction under optimized conditions, yielding exceptional yields [88].

To embrace the chemical process, DFT calculations were performed in the study. The results demonstrated that the diphenyliodonium triflate has a feasible energy barrier of 21.5 kcal/mol and can be readily converted into a stable iodonium thiolate species. This species can further undergo a C–S bond-forming reductive elimination, providing the sulfide product. As a result of C–S bond formation, the oxidation state of iodine is reduced from +III to +I, causing the loss of hypervalency. This process is extremely exergonic and provides the driving force for the reaction. Additionally, the alternative mechanism of a direct attack of the thiolate nucleophile on the aryl group of the iodonium salt was also investigated. A potential radical-mediated mechanism was evaluated.

**Scheme 35:**
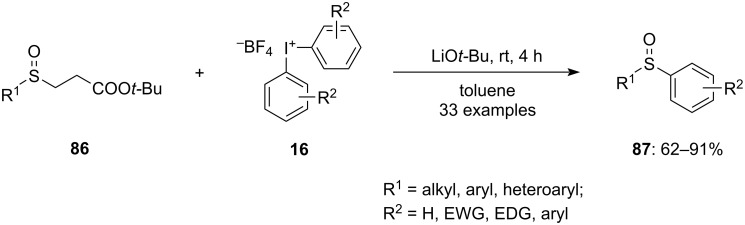
Base-promoted synthesis of diarylsulfoxides **87** via arylation of general sulfinates **86**.

Under mild conditions, a base-promoted arylation of general sulfinates **86** without the need for transition metals was introduced using diaryliodonium salts **16** as an aryl source (Scheme 35). Inspired by these encouraging outcomes, Wang and co-workers looked at the range of substrates that diaryliodonium salts could occupy. Numerous diaryl sulfoxides **87** were synthesized from the respective substrates **86** bearing electron-releasing, electron-neutral, and electron-attracting substituents at various positions of the aryl group. All the tested anions were well-tolerated. Moreover, the rate of productivity of the reaction was not significantly impacted by the steric barrier of the substituents on the sulfonate anions. Further, diaryliodonium salts with electron-releasing substituents at the *para* position of the phenyl groups were good reaction partners. Additionally, a few electron-withdrawing groups also proved to be better coupling partners. The tolerance of unsymmetrical diaryliodonium salts was further verified, resulting in the formation of two distinct products. Notably, the yield of the product with the aryl-containing bulky group was significantly higher compared to the smaller substituents. The arylation of sulfonate anions by transfer of the aryl group with an iPr substituent gave good results on using OTf as the counter anion instead of BF_4_ [89].

**Scheme 36:**
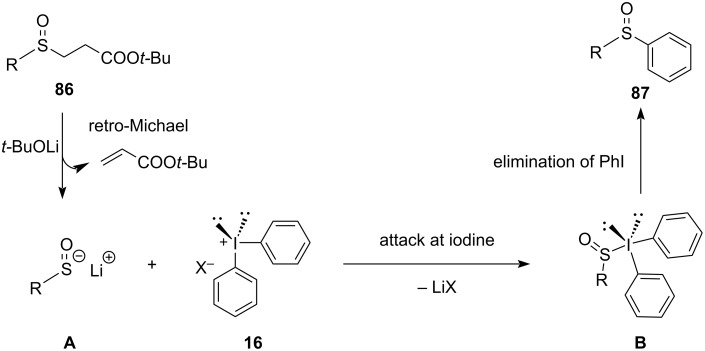
Plausible mechanism for the arylation of sulfinates **86** via sulfenates **A** to give diaryl sulfoxides **87**.

With regard to the mechanism, the base-mediated deprotonation of substrates **86** produces the corresponding ester enolate. This enolate undergoes a retro-Michael reaction, generating sulfenate anion **A**. The sulfenate anion **A** then nucleophilically attacks the diaryliodonium salts **16**, forming hypervalent iodine intermediates **B**. Finally, reductive elimination of intermediate **C** yields the desired sulfoxides **87** (Scheme 36).

**Scheme 37:**
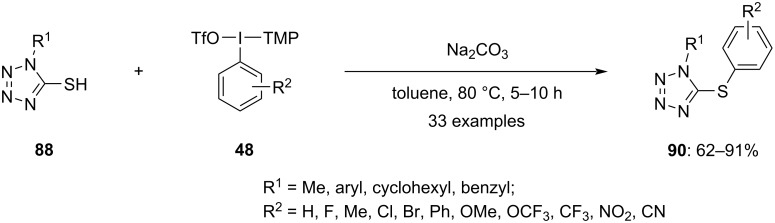
*S*-Arylation reactions of aryl or heterocyclic thiols **88**.

Recently, a detailed study by Thakur and group on the metal-free arylation of tetrazole-5-thiols **88**, exploring various substrate scopes under optimized conditions was conducted (Scheme 37) [90]. The findings indicated a broad functional group tolerance to steric hindrance and electronic factors within this protocol. Investigating the chemoselectivity of unsymmetrical diaryliodonium salts, the researchers noted their comparable electronic factors. Interestingly, the simple phenyl group was transferred easily rather than the phenyl with substituents, when an unsymmetrical diaryliodonium salt was employed. If both phenyls were substituted, then the phenyl ring bearing electron-withdrawing groups led to minor products, with other compounds becoming dominant. Further, replacing one aryl group of the diaryliodonium salt with thiophene favored the formation of phenylated products as the major outcome. Additionally, mercaptoazoles were found to be compatible with this protocol, expanding its applicability to include these compounds.

**Scheme 38:**
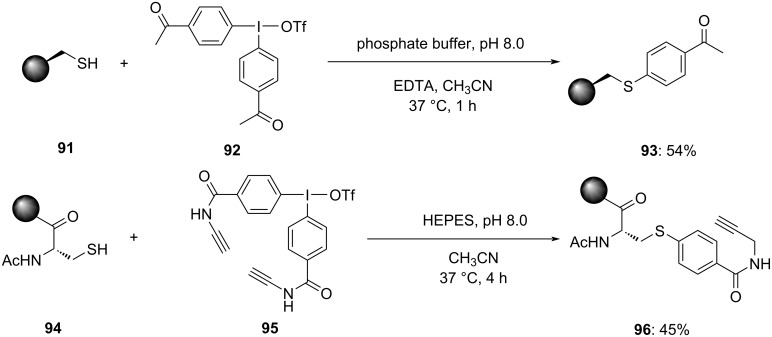
Site-selective *S*-arylation reactions of cysteine thiol groups in **91** and **94** in the presence of diaryliodonium salts **92** and **95** as aryl source.

The functionalization of peptides and proteins plays a vital role in the development of therapeutics, particularly in antibody–drug conjugates (ADCs). Cysteine (Cys) holds a special place in this context due to the distinctive nucleophilicity of its thiol side chain. The cysteine thiol group offers a reactive handle for site-selective modifications, allowing for the attachment of various functional entities. In 2021, Byrne et al. published an impressive report detailing a novel protocol for the chemoselective late-stage variation of proteins and peptides at cysteine residues **91** and **94** in an aqueous buffer in the presence of suitably functionalized diaryliodonium salts **92** and **95** (Scheme 38) [91]. This method manifests the synthesis of stable thioether-linked synthetic conjugates **93** and **96** displaying its efficacy via the alteration of the affibody zEGFR and the histone protein H2A. The procedure involved synthesizing the diaryliodonium salt and appraising the proficiency of oxime ligation chemistry on the histone H2A protein. The protein was demonstrated as the T120C mutant via site-directed mutagenesis in *Escherichia coli* and decontaminated by HPLC. A notable reconciliation was changing the aqueous buffer from HEPES to phosphate owing to side-product generation during the arylation in HEPES. The reaction of H2A with the diaryliodonium salt in phosphate buffer resulted in the expected arylated conjugate in an hour with a maximum of 98% conversion, yielding sulfur arylated product in 54% isolated yield after purification by HPLC.

Additionally, the functionalization of the ketone with a protein conjugate was effectively done with various hydroxylamines with the assistance of the nucleophilic catalyst 5-methoxyanthranilic acid. The resultant histone protein conjugates were functionalized with TAMRA, biotin and the cell-penetrating peptide 'penetratin' through oxime ligation, achieving high conversions (83–93%) in 1–20 hours. Purification by HPLC yielded the isolated oxime conjugates in excellent amounts. This methodology presents a promising approach for the late-stage variation of proteins and peptides, offering versatility and efficiency in aqueous environments.

**Scheme 39:**
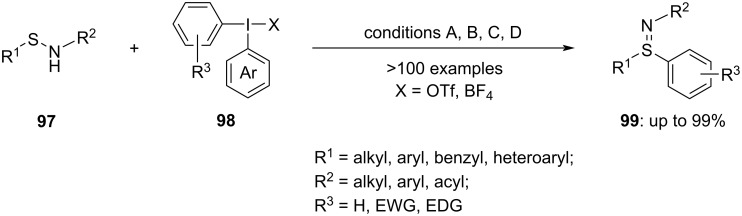
The selective *S*-arylation of sulfenamides **97** using diphenyliodonium salts **98**.

In addition to thiols, sulfoxides and sulfilimines have received significant attention in chemical biology and synthetic chemistry due to their versatile properties. Traditional approaches to sulfilimine synthesis typically include the oxidative imination of sulfides, often relying on transition-metal catalysts, which can present limitations. However, a more efficient method for synthesizing sulfilimines emerged in 2023, involving the selective *S*-arylation of sulfenamides **97** with diaryliodonium salts **98** at ambient temperature in the presence of air (Scheme 39). These innovative approaches offer promising alternatives for sulfilimine synthesis, potentially overcoming some of the drawbacks associated with traditional methods. The choice of base used in the reaction can influence both the reaction duration and the yield. When the reaction was performed in acetonitrile in the presence of Cs_2_CO_3_ as a base at room temperature for 18 h, the yield was quite high (conditions A) [92]. Meanwhile, a similar yield was obtained in 2 h, when NaOH (conditions B) was used as a base (R^2^ = acyl). Upon moving to other substrates (R^2^ = aryl), the yield dropped significantly. By replacing the strong base with K_2_CO_3_, (conditions C) the product yield increased [93]. Similarly, the arylation of sulfonamides can also be achieved by using *t*-BuONa in toluene for an hour at room temperature in the presence of air (conditions D) [94].

**Scheme 40:**
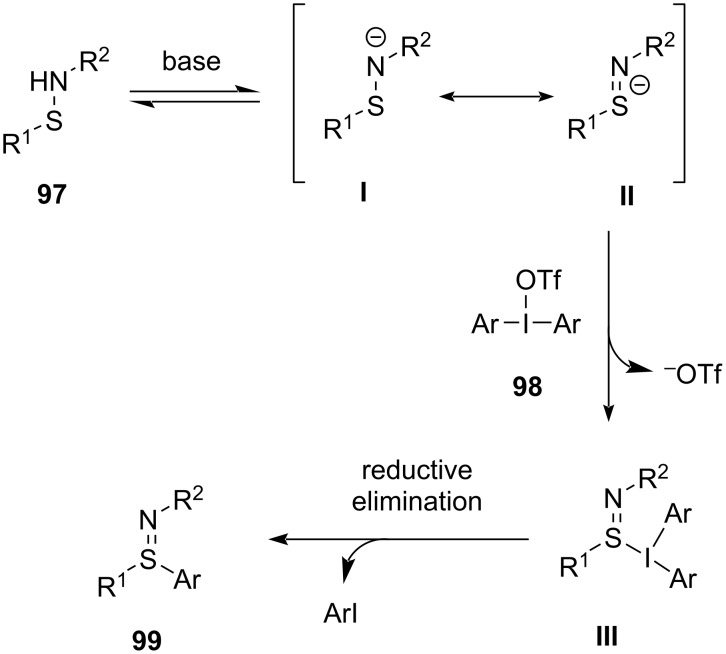
Plausible mechanism for the synthesis of sulfilimines **99**.

The mechanism of the reaction involves formation of anionic intermediates **I** and **II** by the action of the base on *N*-sulfenamides **97**. Both, the divalent N-centered anion intermediate **I** and S-centered anionic intermediate **II** are the resonating structures which further react with the diaryliodonium salt to obtain intermediate **III** along with the elimination of triflate. Finally, intermediate **III** undergoes reductive elimination to produce the desired sulfilimine **99** (Scheme 40) [92].

**Scheme 41:**
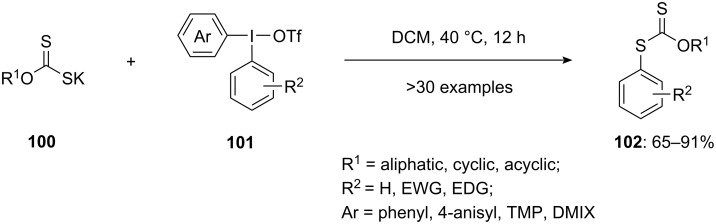
Synthesis of *S*-arylxanthates **102** by reacting DAIS **101** with potassium alkyl xanthates **100**.

Synthesizing *S*-aryl xanthates through transition-metal-catalyzed or S_N_Ar reactions presents challenges due to potential additional transformations occurring under the reaction conditions. However, employing diaryliodonium salts **101** for the *S*-arylation of potassium *O*-alkyl xanthates **100** offers a simpler approach [95]. This method operates under mild conditions, facilitating the creation of substituted *S*-aryl xanthates **102** (Scheme 41). Utilizing diaryliodonium salts for the arylation of xanthate anions provides a pathway for the reaction to proceed under gentle conditions. This prevents the subsequent transformation of the obtained *S*-aryl xanthates, thereby facilitating their isolation with satisfactory to excellent yields. During the assessment of the reaction's versatility, first variously substituted (4-anisyl)(aryl)iodonium salts were explored. The incorporation of the 4-anisyl group was expected to enhance selectivity for transferring unsubstituted phenyl groups. As anticipated, utilizing this component under optimized conditions led to highly selective reactions. This generated a diverse range of substituted *S*-aryl xanthates, with yields spanning from 65% to 91%. The promising biological properties of the *S*-aryl *O*-alkyl xanthates, makes them desirable targets for synthesis, and thus the exploration of the reaction's scope was broadened to encompass xanthate salts with diverse *O*-alkyl substituents.

**Figure 2:**
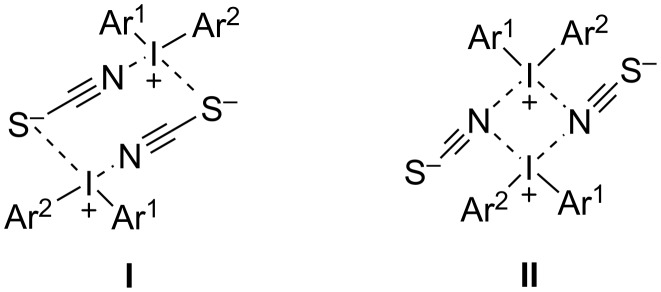
Structured of the 8-membered and 4-membered heterotetramer **I** and **II**.

Another potassium salt, potassium thiocyanate (**104**), was utilized as a sulfur source, with the sulfur serving as the anionic counterpart of diaryliodonium salts **105**. The subsequent arylation involved the arylating action of the counter cationic part of diaryliodonium salts **103** on the anionic thiocyanate component. These arylation studies primarily concentrated on iodonium salts represented by 8-membered cyclic heterotetramer **I** and 4-membered cyclic heterotetramer structures **II**, depicted in Figure 2. The research benefited from the strong regioselectivity of TMP-substituted iodonium cations in nucleophilic substitution reactions.

**Scheme 42:**
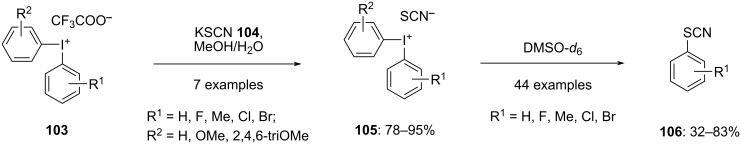
*S*-Arylation by diaryliodonium cations **103** using KSCN (**104**) as a sulfur source.

The reactions were conducted at 100 °C, both in the solid-state and in DMSO-*d*_6_ solution, for comparison. The conventional *S*-arylation reactions worked very well in DMSO-*d*_6_ solution yielding the aryl-SCN products **106** (Scheme 42). However, a significant difference was observed in the solid-state reactions, particularly with iodonium salts exhibiting 4-membered cyclic heterotetrameric structural motifs. Here, the major product was the *N*-arylation compounds ArNCS, contrasting with the predominantly *S*-arylation products obtained in DMSO-*d*_6_ solution reactions [96].

**Scheme 43:**
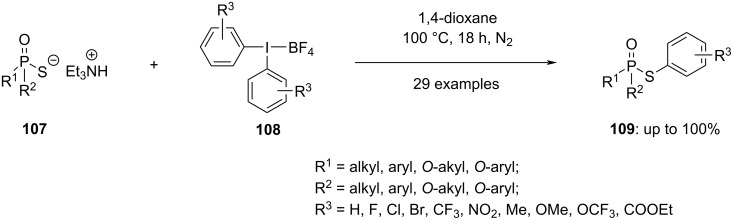
*S*-Arylation of phosphorothioate diesters **107** through the utilization of diaryliodonium salts **108**.

Sarkar and Kalek showcased a novel technique involving the *S*-arylation of phosphorothioate diesters **107** through the utilization of diaryliodonium salts **108** (Scheme 43) [97]. This approach enables the facile synthesis of a diverse array of *S*-aryl phosphorothioates **109**, encompassing complex molecules, and various other organophosphorus compounds that are arylated at a chalcogen. Notably, the reaction retains the stereochemistry at the phosphorus atom, thereby providing a simple method for the synthesis of P-chiral products.

**Scheme 44:**
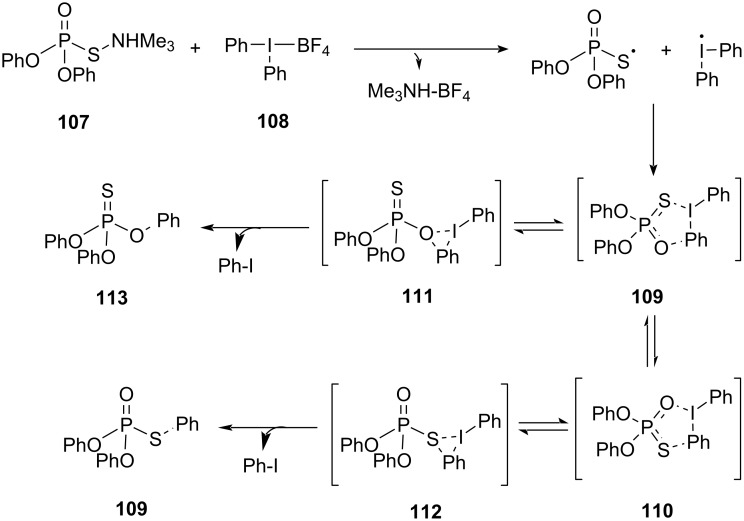
Transfer of the aryl group from the hypervalent iodonium salt **108** to phosphorothioate diester **107**.

Computational studies employing DFT calculations were conducted to delve into the mechanism of the reaction. Specifically, the focus was on understanding the intricacies of the S–Ar bond formation and providing a rationale for the selectivity favoring *S*-arylation over *O*-arylation. Despite numerous attempts, a direct nucleophilic attack of a phosphorothioate on the phenyl ring of the diphenyliodonium salt by substituting an iodine-based leaving group in an S_N_2 reaction mechanism, could not be located. The results showed the phosphorothioate gets attached in the inner coordination sphere of iodine giving intermediates with either P–S–I or P–O–I linkages (**109** and **110**, respectively). Both the intermediates **109** and **110** were energetically similar to each other, suggesting an equilibrium between these species. Homolytic cleavage of the S/O–I bond in **109**/**110** was found to be highly endergonic, excluding the radical pathway of the reaction. This was supported by a study including DPE and TEMPO. The intermediates obtained in the reaction could undergo aryl transfer by two different pathways, which included transition states with three- or five-membered rings (**111** and **112**, respectively) leading to *O*- and *S*-arylation products (Scheme 44). The energetically preferred S–Ar forming **112** (from intermediate **110**) and **111** (from intermediate **109**) explained the observed selective *S*-arylation. It was observed that the five-membered rings were favored transition states due to less strain on the ring. The inner sphere mechanism shared similarities with other aryl transfers using hypervalent iodine salts, with the unique presence of the transition state with a five-membered ring (**109** and **110**) attributed to the structural arrangement of the phosphorothioate diester. Computational studies also indicated stereospecific *S*-arylation of P-chiral phosphorothioates, consistent with the experimental observations of retention of configuration at the phosphorus atom throughout the mechanistic pathway.

**Scheme 45:**
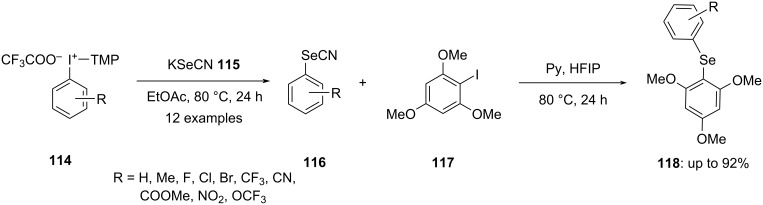
Synthesis of diarylselenides **118** via diarylation of selenocyanate **115**.

Expanding the application of the developed arylation conditions, other P–S nucleophiles and related selenium compounds were investigated. Aryl transfer to the selenium atom of phosphoroselenoates **116** was achieved, though with reduced efficiency. To address this limitation, Radzhbov introduced a novel method for the metal-free diarylation of selenocyanate using trimethoxyphenyl-substituted iodonium salts **114**. This approach facilitated the synthesis of diarylselenides **118** containing electron-donating TMP groups in a two-step reaction without the need for isolating intermediate products (Scheme 45) [98].

To investigate how electron-rich groups influence the arylation, 1,3,5-trimethoxybenzene in the iodonium salts was replaced with 1,3-dimethoxybenzene. Arylation of SeCN^−^ proceeded smoothly in all cases, producing selenocyanides with high selectivity. Surprisingly, when ArSeCN was reacted with 1-iodo-2,6-dimethoxybenzene, it exclusively formed symmetrical diselenides in excellent yields, rather than diarylselanes as expected.

### *P*-Arylation

**Scheme 46:**
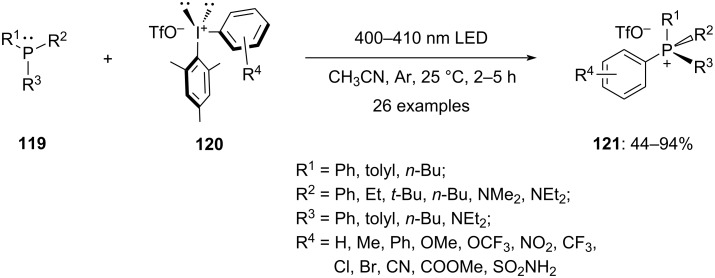
Light-promoted arylation of tertiary phosphines **119** to quaternary phosphonium salts **121** using diaryliodonium salts **120**.

Bugaenko et al. established a light-promoted metal-free and catalyst-free arylation of tertiary phosphines **119** using diaryliodonium triflate salts **120**, yielding quaternary phosphonium salts **121** (Scheme 46). Using this novel protocol, a series of substituted aryl(mesityl)iodonium triflates **120** with varying electronic and steric effects were examined. The triflates bearing either electron-pushing or electron-pulling groups at different positions of the phenyl ring reacted efficiently with excellent selectivity of the aryl group transfer. Moreover, the reaction shows good compatibility across functional groups. Further, aryls with various electronic substituents are transferred for all aryl(mesityl)iodonium salts examined, when the mesityl group is considered to be a "auxiliary" group. Notably, when electron-rich aryls were transferred, the time duration of the reaction was elevated to obtain maximum yields. Since the reaction appears to exhibit electronic preferences based on the observed chemoselectivity, it is recommended that the more electron-poor aryl group is transferred. Additionally, the scope of various tertiary phosphates concluded that changing the predetermined reaction conditions is not required to overcome the steric hindrances. Furthermore, with no indications of the N-quaternization products, the arylation of additional organophosphorus(III) compounds, including phosphinous and phosphonous amides, likewise proceeds fruitfully [99].

Karchava and team expanded the use of hypervalent iodine salts to the arylation of aminophosphorus compounds **122**, resulting in the formation of phosphine oxides **123** through oxidative P–N bond cleavages. This innovative method relies solely on light with wavelength of the visible region as the catalyst to achieve C–P bond formation and tolerates various functional groups.

**Scheme 47:**
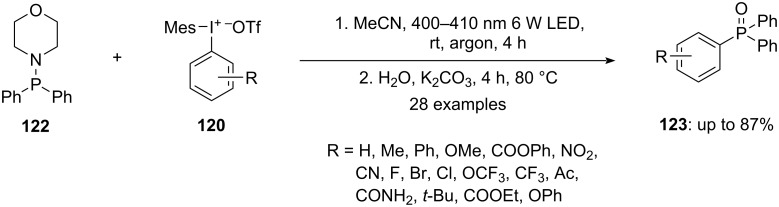
Arylation of aminophosphorus substrate **122** to synthesize phosphine oxides **123** using aryl(mesityl)iodonium salts **120**.

It was observed that on irradiating 4-(diphenylphosphino)morpholine (**122**) dissolved in acetonitrile with blue LED of 400–410 nm wavelength and power of 6 W in the presence of aryl(mesityl)iodonium triflates **120** yielded aminophosphonium salts **123** within 4 h (Scheme 47). The reaction did not require any additive or high temperature and was highly selective as no products were obtained due to *N*-arylation or mesityl transfer. It was noticed that the steric effect had more influence on the selectivity of the aryl transfer in comparison to the electronic effect. The protocol was successful in synthesizing phosphine oxides even in the presence of a substituent at the *ortho* position, demonstrating that the protocol has excellent tolerance towards steric hindrance [100].

### Rearrangement reactions

**Scheme 48:**
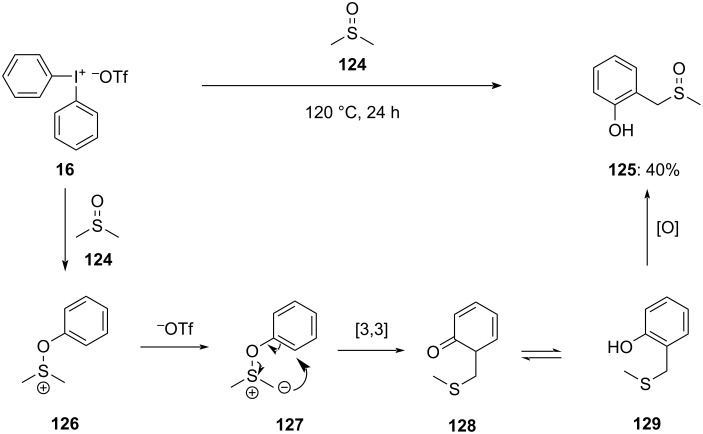
Reaction of diphenyliodonium triflate (**16**) with DMSO (**124**) via thia-Sommelet–Hauser rearrangement.

In 2022, Kepski demonstrated an unexpected arylation phenomenon when diphenyliodonium triflate (**16**) was heated in DMSO. It has been observed that diaryliodonium salts with substitution on phenyl and cyclic DAIS are less prone to react with DMSO. The reaction with DMSO is thought to follow a mechanism similar to the Pummerer and interrupted-Pummerer processes. The reaction starts with the arylation of the oxygen in DMSO yielding sulfonium ion **126**, which further deprotonates to give ylide **127**. Succeeding thia-Sommelet–Hauser rearrangement of ylide **127** affords compound **128**. Rearomatization then yields the product **129**, which immediately oxidizes to sulfoxide **125** in the presence of silica (Scheme 48). Since the 1,4-alkylated phenol is not obtained which indicates that the process involves rearrangement instead of elimination of phenol and subsequent addition [101].

**Scheme 49:**
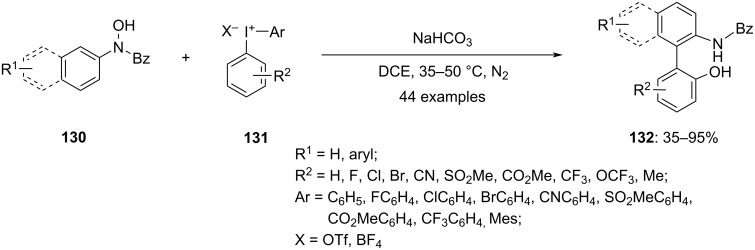
Synthesis of biaryl compounds **132** by reacting diaryliodonium salts **131** with arylhydroxylamines **130** in the presence of a base.

Gao and group observed a similar rearrangement when diaryliodonium salts **131** were treated with arylhydroxylamines **130** in the presence of a base. After optimizing the reaction conditions, they investigated the scope and limitations of this transformation. They found that this transition-metal-free tandem approach could be applied to a wide range of arylhydroxylamines **130** and diaryliodonium salts **131**, yielding diverse highly functionalized biaryl amino alcohol motifs **132** (Scheme 49). They successfully obtained the desired racemic NOBIN-type products in decent to outstanding yields, showcasing excellent regioselectivity. Both the starting material compounds’ **130** and the diaryliodonium salts’ **131** substituents were effectively accommodated under the standard conditions. Notably, this methodology enabled the efficient preparation of biarylamino alcohols with multiple halogens or trifluoromethyl substitutions, which are challenging to access through conventional approaches. Furthermore, the protocol was also suitable for synthesis of naphthyl–phenyl non-*C*_2_-symmetrical biaryls. The transformation was scalable, and the resulting biaryls could be further converted into new heterocycles and atropisomeric biaryl compounds [102].

**Scheme 50:**
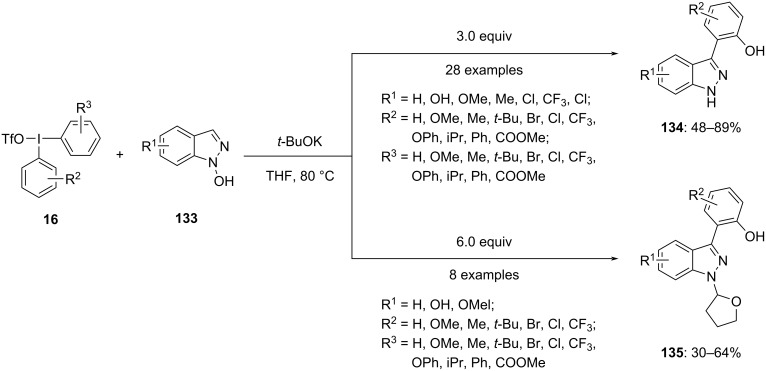
Synthesis of substituted indazoles **134** and **135** from *N*-hydroxyindazoles **133**.

Recently, Nie et al. approached a transition-metal-free diaryliodonium salt-mediated protocol to synthesize a series of functionalized *N*-(tetrahydrofuran-2-yl)-3-(2-hydroxyaryl)indazoles **135** and 3-(2-hydroxyaryl)indazoles **134** with moderate to good yields. The reported method encompasses a radical *O*-arylation followed by a sequential [3,3]-rearrangement cascade approach starting from *N*-hydroxyindazoles **133** and diaryliodonium salts **16** (Scheme 50). The quantity of diaryliodonium salts added to the reaction strongly influenced the structure of the expected products. The mechanistic study found that both *O*-arylation and N–O bond cleavage proceed via a [3,3]-rearrangement, which involves the transformation of specific groups within the molecules. Furthermore, this rearrangement occurs through a process involving radicals formed between different molecules, known as an intermolecular radical process. The reaction confirmed tolerance towards a number of functional groups, as aldehydes, esters, and halides [103].

## Conclusion

In conclusion, this review emphasises the exceptional selectivity and reactivity of DAIS that facilitate efficient arylation reactions with a diverse range of nucleophiles. This work includes recent advancements in practically simple and scalable methods for the arylation of carbon, nitrogen, sulfur, oxygen, and phosphorus in metal-free conditions. Both symmetrical and unsymmetrical diaryliodonium salts are employed to yield arylated products. Generally, it is observed that in unsymmetrical diaryliodonium salts, the transfer of the aryl group is influenced by its steric and electronic properties, favoring the transfer of the aryl group with lower electron density and less steric hindrance.

## Data Availability

Data sharing is not applicable as no new data was generated or analyzed in this study.
